# Hantavirus in Panama: Twenty Years of Epidemiological Surveillance Experience

**DOI:** 10.3390/v15061395

**Published:** 2023-06-19

**Authors:** Blas Armién, Carlos Muñoz, Hector Cedeño, Jacqueline R. Salazar, Tybbyssay P. Salinas, Publio González, José Trujillo, Deyanira Sánchez, Jamileth Mariñas, Ayvar Hernández, Harmodio Cruz, Leisy Yovany Villarreal, Elba Grimaldo, Samuel González, Heydy Nuñez, Susana Hesse, Fernando Rivera, George Edwards, Ricardo Chong, Ovidio Mendoza, Martín Meza, Milagro Herrera, Rudick Kant, Raul Esquivel, Dora Estripeaut, Demetrio Serracín, Bernardino Denis, Esthefani Robles, Yaxelis Mendoza, Gloria Gonzalez, Felicia Tulloch, Juan Miguel Pascale, Jonathan L. Dunnum, Joseph A. Cook, Anibal G. Armién, Fernando Gracia, Gladys Alicia Guerrero, Itza de Mosca

**Affiliations:** 1 Department of Research in Emerging and Zoonotic Infectious Diseases, Gorgas Memorial Institute of Health Studies, Panama City 0816-02593, Panama; jsalazar@gorgas.gob.pa (J.R.S.); tsalinas@gorgas.gob.pa (T.P.S.); pgonzalez@gorgas.gob.pa (P.G.); demetrioserracin@yahoo.com (D.S.); bernardinodenis@gmail.com (B.D.); emroblesv@gmail.com (E.R.); 2 Sistema Nacional de Investigación (SNI), Secretaria Nacional de Ciencia y Tecnología (SENACYT), Panama City 0816-02852, Panama; destripeaut@gmail.com (D.E.); ygmendoza@gorgas.gob.pa (Y.M.); 3 Department of Epidemiology, Health Region of Los Santos, Ministry of Health, Las Tablas, Los Santos 0816-06812, Panama; cmunoz@minsa.gob.pa; 4 National Department of Epidemiology, Ministry of Health, Panama City 0816-06812, Panama; hcedeno@minsa.gob.pa (H.C.); ibarahona@minsa.gob.pa (I.d.M.); 5 Hospital Gustavo Nelson Collado, Caja de Seguro Social, Chitre, Herrera 0816-06808, Panama; jmtmrt14@cableonda.net (J.T.); shgv@hotmail.com (S.G.); 6 Hospital Rural de Tonosí, Ministerio de Salud, Tonosi, Los Santos 0816-06812, Panama; deyaluly@hotmail.com (D.S.); drajamilethm@gmail.com (J.M.); aihernandezs@minsa.gob.pa (A.H.); harmodiocruzsamaniego@gmail.com (H.C.); leisyyovavillarreal1309@outlook.com (L.Y.V.); 7 Hospital Joaquín Pablo Franco Sayas, Ministry of Health, Las Tablas, Los Santos 0816-06812, Panama; elbaisabelg@gmail.com; 8 Hospital Ezequiel Abadía, Caja de Seguro Social, Soná, Veraguas 0816-06808, Panama; heynunez@css.gob.pa; 9 Department of Epidemiology, Caja de Seguro Social, Santiago, Veraguas 0816-06808, Panama; 10 Hospital Dr. Luis Chicho Fábrega, Ministry of Health, Santiago, Veraguas 0816-06812, Panama; susanahesse@gmail.com (S.H.); friverach9@icloud.com (F.R.); geedwards@minsa.gob.pa (G.E.); 11 Department of Epidemiology, Health Region of Veraguas, Ministry of Health, Santiago, Veraguas 0816-06812, Panama; rmchong@minsa.gob.pa (R.C.); pabloovi29@gmail.com (O.M.); 12 Department of Epidemiology, Health Region of Herrera, Ministry of Health, Chitre, Herrera 0816-06812, Panama; mmeza07@yahoo.com; 13 Hospital Regional Rafael Estevez, Caja de Seguro Social, Aguadulce, Coclé 0816-06808, Panama; miherrera@css.gob.pa; 14 Department of Epidemiology of the Caja de Seguro Social, Panama City 0816-06808, Panama; rkant@css.gob.pa (R.K.); glguerrero@css.gob.pa (G.A.G.); 15 Hospital del Niño José Renal Esquivel, Panama City 0816-00383, Panama; resquivel@hn.sld.pa; 16 Department of Genomics and Proteomics, Gorgas Memorial Institute of Health Studies, Panama City 0816-02593, Panama; ggonzalez@gorgas.gob.pa (G.G.); jmpascale@gorgas.gob.pa (J.M.P.); 17 Hospital Santo Tomás, Panama City 0816-05602, Panama; fetuba2000@yahoo.com (F.T.); fegra@medicospaitilla.com (F.G.); 18 Department of Biology and Museum of Southwestern Biology, University of New Mexico, Albuquerque, NM 87131, USA; jldunnum@unm.edu (J.L.D.); cookjose@unm.edu (J.A.C.); 19 California Animal Health & Food Safety Laboratory System (CAHFS), School of Veterinary Medicine, University of California, Davis, CA 95616, USA; agarmien@ucdavis.edu

**Keywords:** *Choclo orthohantavirus*, hantavirus disease, hantavirus pulmonary syndrome, *Oligoryzomys costaricensis* (*=fulvescens*), hantavirus fever, epidemiologic surveillance, One Health

## Abstract

Twenty years have passed since the emergence of hantavirus zoonosis in Panama at the beginning of this millennium. We provide an overview of epidemiological surveillance of hantavirus disease (hantavirus pulmonary syndrome and hantavirus fever) during the period 1999–2019 by including all reported and confirmed cases according to the case definition established by the health authority. Our findings reveal that hantavirus disease is a low-frequency disease, affecting primarily young people, with a relatively low case-fatality rate compared to other hantaviruses in the Americas (e.g., ANDV and SNV). It presents an annual variation with peaks every 4–5 years and an interannual variation influenced by agricultural activities. Hantavirus disease is endemic in about 27% of Panama, which corresponds to agroecological conditions that favor the population dynamics of the rodent host, *Oligoryzomys costaricensis* and the virus (*Choclo orthohantavirus*) responsible for hantavirus disease. However, this does not rule out the existence of other endemic areas to be characterized. Undoubtedly, decentralization of the laboratory test and dissemination of evidence-based surveillance guidelines and regulations have standardized and improved diagnosis, notification at the level of the primary care system, and management in intensive care units nationwide.

## 1. Introduction

The first outbreak of hantavirus pulmonary syndrome (HPS) and hantavirus disease (HD) was described in the United States of America in 1993 [1]. This disease was associated with *Sin Nombre orthohantavirus* (SNV), which is the prototype virus for the Americas and whose primary reservoir is *Peromyscus maniculatus* [2]. Newly developed molecular technology and access to biorepository tools have allowed the diagnosis and identification of many other orthohantaviruses in America over the past three decades from a taxonomically diverse group (Rodentia, Chiroptera, Eulipotyphla) of reservoir mammals [3,4]. Viral infection in humans occurs mainly through the inhalation of aerosols from the excreta of reservoir rodents. Person-to-person transmission of *Andes orthohantavirus* (ANDV) has been reported in Chile and Argentina [5,6,7,8,9], but a recent review of the specific ANDV cases now casts doubt on this possibility [10]. To date, HPS outbreaks have been most closely associated with the population dynamics and ecology of rodent hosts [11].

Currently, 25 pathogens have been identified which belong to the Family Hantaviridae of the Order Bunyaviridae [12]. Near the end of 1999 and early 2000, the first outbreak of HPS was identified in Panama and found to be caused by the subsequently described *Choclo orthohantavirus* (CHOV), which occurs primarily in *Oligoryzomys costaricensis* (=*fulvescens*) [13,14,15,16]. Anthropogenic activities are likely the underlying cause for the emergence of this zoonosis due to the substantial alteration of natural areas near towns for agricultural production and livestock grazing, which are the basis of the economy and human subsistence in the central provinces of Panama. Studies that characterized the ecology of *Oligoryzomys costaricensis* support this hypothesis and reveal that this species is not only widely distributed in the lowlands of west-central Panama but also thrives in the agricultural fields and pastures of the region [17].

Twenty years have passed since the emergence of this zoonosis in Panama. Herein, we aim to describe this experience from an epidemiological perspective to begin to understand the demographic characteristics and spectrum of the disease, including incidence, case-fatality rate, and mortality rates across both geographic and temporal scales. During this two-decade period, new evidence led to adjustments to the case definition established in 2000 by the Health Authority [18,19]. The objective of this work is to describe the findings of epidemiological surveillance of the hantavirus disease (HD = HPS + hantavirus fever) during the period 1999–2019 in Panama and to strengthen proactive early detection, control, prevention, and management of HPS.

## 2. Materials and Methods

### 2.1. Case Definition

All cases reported nationally from 1999 to 2019 that met the case definition of hantavirus disease established by the Ministry of Health of Panama [13,20] were included. According to the 2016 Panama hantavirus disease management guide [19], a suspected case was defined as any person presenting fever (temperature > 38.5 °C), myalgia, headache, accompanied or not by gastrointestinal symptoms, who also has a history of exposure to risky activities or wild rodents approximately 1–6 weeks prior to symptom onset or who has visited or is from an endemic area for Hantavirus disease and who has any of the following conditions: thrombocytopenia; hemoconcentration; chest X-ray with unilateral or bilateral interstitial infiltrate; a previously asymptomatic person who presents a picture of respiratory distress (ARDS) for no reason to explain it; or unexplained respiratory symptoms, resulting in death and autopsy showing non-cardiogenic pulmonary edema, with no specific cause identifiable by the laboratory. The confirmed case corresponds to a suspected case that has been confirmed by the laboratory in which an acute infection by hantavirus is demonstrated (detection of IgM antibody against hantavirus or detection of a hantavirus-specific RNA sequence by PCR). Surviving cases or deaths diagnosed with hantavirus disease was confirmed through serologic and molecular biology tests using previously described methodology [18,19].

### 2.2. Data Collection

In 2000, the National Department of Epidemiology of the Ministry of Health, together with the Social Security Fund and the Gorgas Memorial Institute for Health Studies, detailed the data collection instruments and the notification system and mechanisms for cases, research field tests, and requests for analysis of laboratory samples for the diagnosis of hantavirus disease. Based on these protocols, a database was prepared using information from the notification form by the National Department of Epidemiology of the Ministry of Health.

The clinical-epidemiological information was obtained from the epidemiological surveillance notification form [19]. The variables included consisted of sex, age, address (township, district, and province), date of onset of symptoms, and clinical spectrum of HD (i.e., hantavirus fever (HF) and HPS (mild to moderate and severe)) (Supplementary Figure S1a,b). Demographic information was available from the National Institute of Statistics and Census (www.inec.gob.pa, accessed on 19 January 2023).

### 2.3. Spatial Distribution

**Figure 1:**
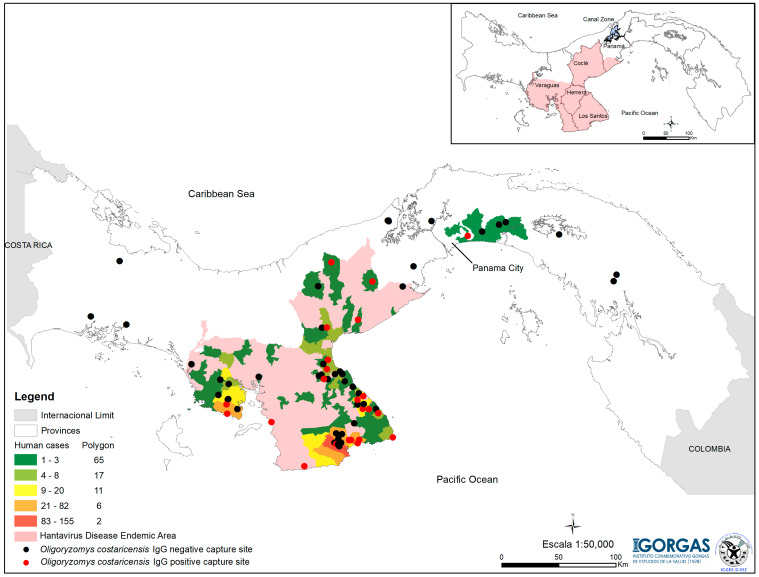
Distribution of hantavirus disease cases confirmed by ‘corregimiento’ (townships). The area highlighted in pink includes the corregimientos where cases of acute illness were detected and coincides with the capture sites of *Oligoryzomys costaricensis* in which positive individuals for *Choclo orthohantavirus* (red dots) have been detected [17,21]. The corregimientos with a higher number of cases are highlighted in yellow, orange, and red.

The human cases of hantavirus diseases and the capture sites for *Oligoryzomys costaricensis* were georeferenced using the Datum UTM, WGS 1984, with ArcMap software from ArcGis v. 10.7 (ESRI 2019) used to generate distribution maps (Figure 1).

We used a base map of Panama with a scale of 1/50,000, to which we added the ‘corregimientos’ (townships) layer and linked the numbers of HD cases in humans with the properties of this existing layer; then, we created a color gradient that represents the number of positive people by corregimientos to indicate the endemic areas of the disease. The points show the coordinates of the capture sites of the rodent reservoir of CHOV, *Oligoryzomys costaricensis*. (see Figure 1).

### 2.4. Statistical Analyses

Continuous and categorical variables were analyzed in EPIINFO (Centers for Disease Control and Prevention, Atlanta, GA; Version: 7.2.4.0) and RStudio (2023.03.1 Build 4) and then assessed using parametric and nonparametric techniques. The following five rates were estimated: (a) Specific case-fatality rate using the total annual HPS deaths among the total HPS cases diagnosed and confirmed during the year in which the cases occurred; (b) Hantavirus disease case-fatality rate based on the total annual HPS deaths and the total confirmed HD cases during the year in which the cases occurred; (c) HPS cumulative case-fatality rate was estimated using the total number of HPS accumulated deaths from 1999–2019 and the total HPS cases diagnosed during the period 1999–2019; (d) Crude annual incidence rate was estimated using the number of new cases of hantavirus disease during the year among the estimated population at risk as of 1 July of the respective year and (e) Specific HPS mortality rate was estimated using the number of fatal HPS cases per specific year among the estimated population at risk as of 1 July of the respective year (https://www.inec.gob.pa, accessed on 16 April 2023). We evaluated the number of accumulated cases and the mortality rate due to HPS by age group and sex. Comparative tables were built considering the variables age, sex, disease spectrum [18], deaths, and their geographical distribution. We performed a five-year analysis to explore the number of diagnosed cases of HD versus HPS according to age groups and assess statistically significant variables (*p* < 0.05).

## 3. Results

### 3.1. Geographical Distribution

During the 20 years of HD surveillance, 712 cases were tallied, mainly in the central-western region of Panama (see Figure 1). The districts highlighted in yellow accumulated between 9 and 20 cases followed by those highlighted in orange with 21–82 cases and the one highlighted in red with more than 83 cases (see Figure 1). The province of Los Santos accumulated 77.4% (n = 550) of the cases, followed by Veraguas (n = 84), Coclé (n = 49) and Herrera (n = 22), which combined constitute the endemic area for HD in Panama (Table 1).

In this area, the rodent reservoir (*O. costaricensis*) was regularly captured, and the CHOV is known to circulate (Figure 1).

In the province of Panama, seven cases of HPS were reported—four men and three women. The first case was reported in 2000 and was within the endemic area (Figure 1, Supplementary Figure S2). The other six cases were diagnosed between 2009 and 2014; five were located in the capital (Panama City) and one to the east of the capital (Supplementary Figure S2). Three women, aged 17, 25 and 56, died. The first two died at home in 2009, and the diagnosis was confirmed by RT-PCR/Nested PCR [18] of tissue samples. In addition, rodents were captured in their homes or nearby surroundings, and these locations met the agroecological risk conditions of pastures and rice crops. However, none of the 25 *Oligoryzomys costaricensis* captured was positive for the detection of IgG antibodies against CHOV or detection of CHOV viral particles by RT-PCR/Nested PCR. The other cases had no clear exposure histories and may have acquired the infection during their travels to the endemic area described previously (Supplementary Figure S2).

### 3.2. Demographic Characteristics and Disease Spectrum

**Figure 2:**
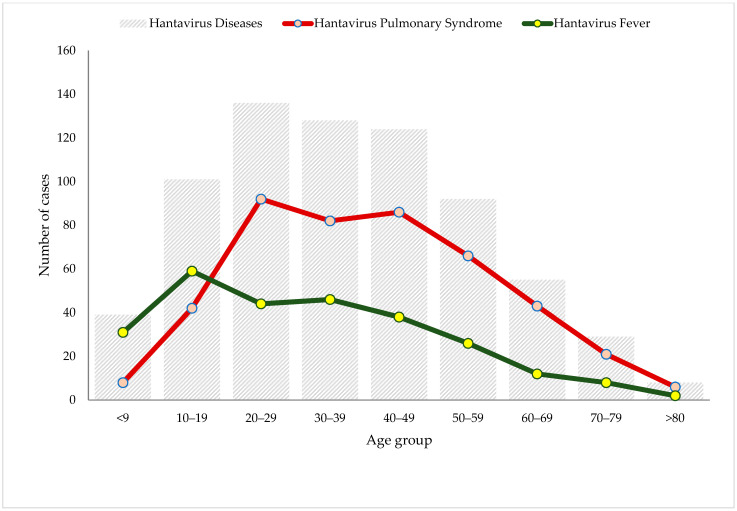
Hantavirus disease cases reported by age group in Panama during 1999–2019.

Surveillance of HD indicates that 37% (266) of the Panamanian cases are HF and 63% (446) are cases of HPS. About 12% of HPS cases were clinically classified as moderate, 33% were severe cases, and the vast majority were mild HPS. Further, 2 of the moderate cases and 54 of the severe cases died, a statistically significant difference (X^2^ = 19.37; *p* < 0.0001) (Supplementary Figure S1a). The mean age of the cases in the areas considered endemic to HD was 37.2 (±18.5) years, varying between 36.3 (±19.0) years in the province of Los Santos and 45.7 (±13.5) years in the province of Herrera (Table 1, Figure 1). Of the total cases of HD, 16% (117/712) were under 18 years of age; of these, 68% (79/117) suffered from HF (Table 1). Men were affected more, with 56% (n = 402) of the total cases of HD, with the male:female ratio at 1.3:1 (Table 1). In HPS cases (n = 446), the number of cases was also higher in men (n = 233) than women (n = 213), although the male:female ratio was lower, 1.09:1. Of the 56 deceased that were diagnosed with HPS, more were women (59%; n = 33) than men (41%; n = 23), but this difference was not statistically significant (OR = 1.67, 95% CI = 0.95–2.95, *p* = 0.0996). The mean age in the cases of HF (31.4 ± 18.9 years) compared to those who suffered HPS (40.6 ± 17.5 years) was lower in the former. This difference was statistically significant (*p* < 0.001 by Kruskal–Wallis test) (Figure 2).

**Figure 3:**
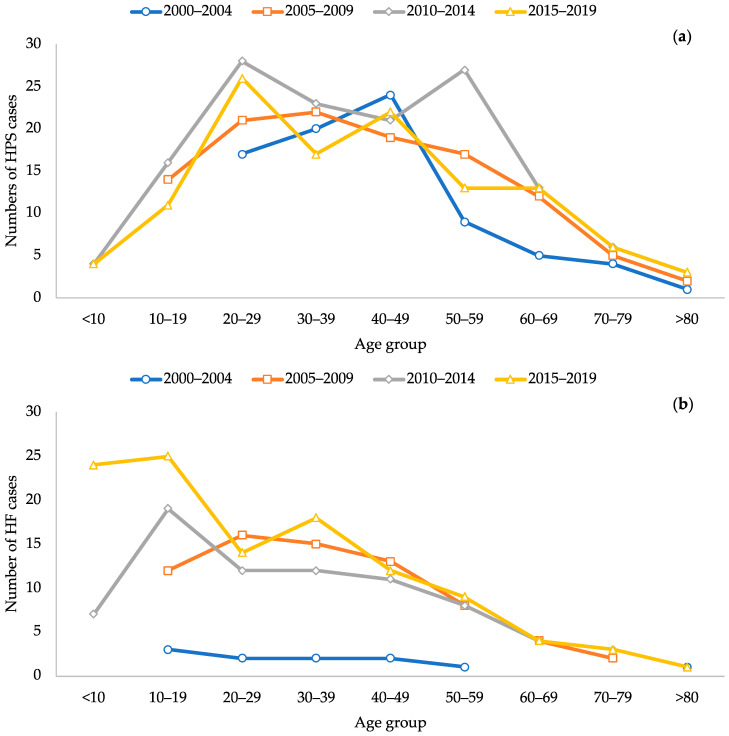
Cases of hantavirus disease reported per five-year period in Panama, during 2000–2019. (**a**) Hantavirus Pulmonary Syndrome; (**b**) Hantavirus fever.

During the 2000–2004 five-year period, HPS cases occurred only in adults between 20 and 80 years of age, but for the 2005–2009 period, there were cases in patients between 10 and 19 years of age. For the periods 2010–2014 and 2015–2019, cases also occurred in patients between 1 and 9 years of age (Table 1, Figure 3a).

According to this analysis, the incidence of HPS cases increased with age, with the majority occurring in those between 20 and 49 years of age, then decreasing after 50 years of age. HF cases occurred mainly among those under 10 years of age up to 30 years of age (Figure 3b). In addition, during the surveillance period, both HPS and HF cases were detected in people under 50 years of age (Figure 3a and Figure 3b, respectively).

### 3.3. Temporal Distribution and Rates of Incidence, Case-Fatality Rate and Mortality

**Figure 4:**
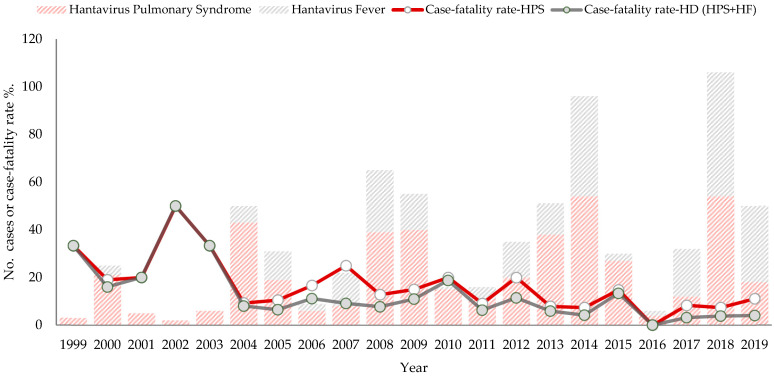
Number of hantavirus disease cases and case fatality rate (HPS, HD) per year, 1999–2019.

HD outbreaks occur every 4–5 years. The earliest cases of HF were reported during the first outbreak in 2000, and then again after 2004, reporting increased. The years with the fewest HD cases occurred between 2001 and 2003 and then in 2016, when just two cases of HF and four cases of mild HPS were reported. The years with the highest reported HD cases occurred in 2014 and 2018. The average annual number of HD cases is 34, with a range of 2–105 cases (Figure 4).

**Figure 5:**
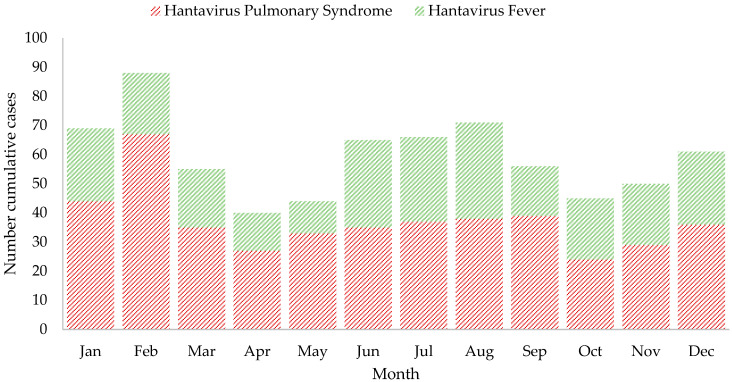
Number of cumulative cases per month of hantavirus disease in Panama, 1999–2019.

The crude annual incidence rate of the disease ranged from 0.10 to 2.52 per 100,000 inhabitants. The overall case-fatality rate due to HPS was 12.6% (56/446), which varied from 7.3% to 50.0% during the years 1999–2019. However, the accumulated case-fatality rate of the hantavirus disease was 7.9% (56/712) and varied from 3% to 50% (Table 1, Figure 4). After 2004, the case-fatality rate for both estimates has a slight tendency to decrease, contrary to the notification of cases that had a tendency to increase. This downward trend in the case fatality rate of HPS and HD throughout the study period was statistically significant (*p* = 0.043 and *p* = 0.0009, respectively, by the Cochran–Armitage trend test) (Figure 4). Except for 2016, a year without deaths, the range was 1–6 deaths per year. The specific mortality rate for HPS was 0.02 to 0.19 per 100,000 inhabitants. HD cases occur in any month of the year, but the highest number of accumulated cases occurred in January to March, which corresponds to the dry season, and in June to September, the rainy season (Figure 5).

**Figure 6:**
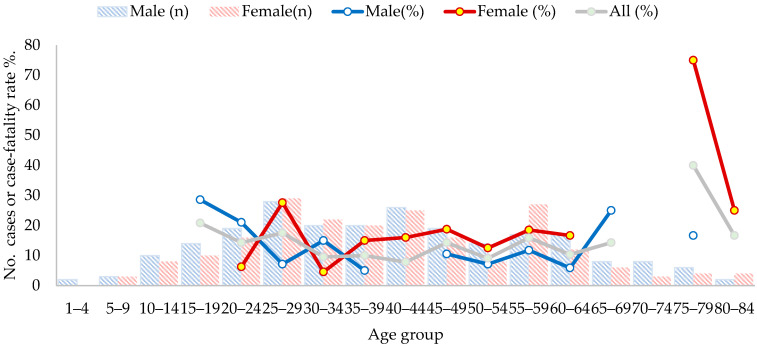
The number of cases (bars) of and case-fatality rate (line) for hantavirus pulmonary syndrome by age group and sex, Panama, 1999–2019.

No deaths were detected in children under 14 years of age during this surveillance period. Deaths were registered at similar rates in individuals from 15 to 69 years of age but were higher above 75 years of age. When the case-fatality rate is broken down by sex, it is slightly higher among women than among men (Figure 6, Table 1).

## 4. Discussion

This first description of the epidemiological surveillance of HD summarizes the period 20 years after the initial outbreak in late 1999/early 2000 [22]. Although the initial cases were described in the province of Los Santos [13], the cases later extended to other neighboring provinces to cover an area of approximately 20,000 km^2^ in the west-central region of Panama. This is supported by the fact that the notification of HD cases has been carried out constantly in that region, where agroecological and housing conditions are prime for CHOV emergence [15,17,18,23]. CHOV has been identified and sequenced both in samples from acute cases of hantavirus disease and from *Oligoryzomys costaricensis* [14,18,24]. Although another *Orthohantavirus* (*Calabazo* virus [14]) has been described from Panama and also occurs in a grassland-associated sigmodontine rodent (*Zygodontomys brevicauda*), to date only CHOV has been associated with HPS. In previous studies, phylogenetic analyses have shown that the nucleotide sequences of the S segment obtained in HPS cases in Panama correspond to CHOV [21,24,25].

Even though seven cases of HPS were reported in the province of Panama, only two met the conditions required to capture rodents and characterize potential risk factors. In those two instances, large numbers of *Oligoryzomys costaricensis* were captured, but none of them were positive in 2009. Between 2011 and 2014, during systematic fieldwork at the national level, 15 *Oligoryzomys costaricensis* were captured in the Tocumen area, and CHOV sequences were identified in 3 of them [21]. However, those captures were more than 10 km from the two reported cases (Figure 1, Supplementary Figure S2). These cases may be autochthonous, and systematic field studies will be necessary to test this possibility [26].

Outside the endemic area described and Panama City, where cases have been reported, *Oligoryzomys costaricensis* has been captured to the west, east and on both sides in the canal area, but CHOV was not detected by serology or PCR sequencing (Figure 1, Supplementary Figure S2) [21]. However, our epidemiological surveillance of HD based on >200 samples collected between 2010 and 2019 from these areas indicated that all samples were negative by both serology and molecular biology. Nevertheless, in both scenarios, the circulation of the CHOV cannot be ruled out.

To date, Panama is the only Central American country that has reported confirmed cases, with a total of 712 cases in 20 years of epidemiological surveillance of hantavirus disease. In addition to the mandatory notification of HPS [13,20], the epidemiological surveillance system of Panama established the surveillance of febrile patients due to hantavirus based on both published evidence and clinical experience observed in the care of cases [19]. In the Americas, HPS surveillance has been established in the surveillance regulations of multiple countries [27]. However, in Chile, hantavirus disease is classified as HPS and mild hantavirus disease, definitions that are similar to those established in Panama for hantavirus fever [28,29]. In Chile, mild hantavirus disease represents 5.3% [28] of reported cases, while in Panama it ranged from 6.3% to 63.4% between 2004 and 2019 (Figure 4).

When evaluating HD in Panama according to sex, HD cases show a male:female ratio of 1.3:1. However, when we evaluate the male:female ratio of HPS, this ratio is 1.09:1. This contrasts with observations in Chile, Argentina and Paraguay, where the male:female ratio was 2.6:1, 3.6:1, and 8:1, respectively, while in the United States, it was 1.7:1 [30,31,32,33,34].

In Panama, the case-fatality rate from HPS in men (M) versus women (W) was 9.9% and 15.5%, respectively. Case-fatality rate from HPS vary across Chile (M: 33.8%/W: 37.1%), Argentina (M: 32.9%/W: 35.5%), and the United States (M: 33.0%/W: 39.0%) [30,32,33]. Hence, the case-fatality rate from HPS in Panama was much lower for both men and women when compared to Chile, Argentina and the United States. Although it was slightly higher in women in all, these differences were not statistically significant.

Hantavirus cases occur every month of the year, but the fluctuation in the number of HD cases is influenced by agricultural activity. In the region, this activity includes preparation, sowing, cleaning, harvesting, drying, transfer and crop storage, which are carried out twice a year depending on the type of crop. The first sowing of the year is carried out in April or May, depending on the region, and the first harvest begins in July and lasts until September. During this same period, a second planting or crop rotation usually occurs. The second harvest begins in November and extends until January or February of the following year. Those activities increase human exposure, either directly during field work or due to increased rodent populations near houses that are proximate to cultivated fields and pastures [17]. Cases occur primarily from April to September, when there is a higher risk of exposure. Cases tended to decrease in October but increase again in November through February, which is the important harvest period where there is substantial grain in the crop fields, probably facilitating the increase in the rodent population [35]. Longitudinal studies are needed to assess the dynamics of the reservoir host population associated with increases in HD cases and test this hypothesis.

HPS in Panama occurs at a relatively low frequency compared to diseases produced by other zoonotic or vector-borne pathogens in Panama (e.g., Malaria, Leishmaniasis, Dengue; [36,37,38,39]). Still HD due to CHOV in Panama occurs at a higher incidence rate than SNV in the USA. Between 1993 and 2009, HD due to SNV in the United States varied from 0.04 to 0.19 cases per million people [30]; in Panama, HD was from 0.63 to 14.05 cases per million people based on a National Institute of Statistics and Census of Panama.

The case-fatality rate of HPS is very different from that observed in association with SNV or ANDV. In the United States, the annual SNV case-fatality rate is 35% [30]. In Chile, the annual case-fatality rate varies from 20 to 35% of HPS cases due to ANDV [33,34]. Although several viruses associated with *Orthohantavirus* circulate in Argentina, the case-fatality rate varies from 17 to 40% per year [32]. In Paraguay, HPS is mostly associated with the circulation of *Laguna Negra orthohantavirus*, whose accumulated case-fatality rate for the period 2013–2020 is 21%, ranging from 9.1% to 50% [31]. In Panama, the accumulated case-fatality rate is 12.4%, varying from 7.4% to 50.0% per year. When comparing the case-fatality rate caused by a single virus as the cause of HPS cases, the case-fatality rate in Panama is one of the lowest. In addition, since CHOV seems to be less pathogenic than other orthohantaviruses described above, the interventions of the Ministry of Health of Panama may have contributed to mitigating the impact. This suggests that it could be due to the low pathogenicity of the virus or genetic factors inherent to individuals, coupled with high prevalence rates in the studies carried out [40,41,42]. It is noteworthy that 72% (HF = 266 + HPS-mild = 249/712) of the cases of hantavirus disease have a fundamentally mild presentation (Supplementary Figure S3).

An important aspect to highlight is that between 2006 and 2009, the number of cases gradually improved with the decentralization of the diagnostic test Strip Immunoblot Assay (SIA) [43] in the health regions of Los Santos and Veraguas. As of 2011, the ELISA test (Hantavirus IgM DxSelect™ from Focus Diagnostics, Cypress, CA, USA) was decentralized. Confirmation by SIA and RT-PCR/Nested PCR was maintained at the ICGES, located in Panama City. The training of health personnel, combined with health promotion in rural communities by the Ministry of Health, the Social Security Fund, and the Gorgas Memorial Institute for Health Studies, was persistent during 2000–2013. The management guide and regulations for epidemiological surveillance of hantavirus disease [19] were released in 2014. This strengthened the notification capacity of health personnel at the national level, with particular emphasis on the endemic regions where suspicion should be maintained in cases that present signs and similar symptoms as other pathologies such as leptospirosis, dengue, rickettsiosis and, more recently, COVID-19 (Supplementary Figure S1) [25,36,44,45,46]. This translated into increased epidemiological notification and documentation of cases observed in the time series. Between 2000 and 2006, surveillance focused on notification of HPS cases. As of 2010–2011, cases of HF were already included [18]. Between the years 2001 and 2003, HD was likely underreported; however, in 2016, with improved surveillance, only six cases of HD occurred, of which four were HPS. Environmental factors and anthropogenic activities may have lowered rodent populations that year, effectively decreasing cases in the endemic region of Panama [35].

Surveillance of this disease remains difficult. Among challenges, we note that there is likely significant underreporting in the provinces of Herrera, Coclé and Veraguas, in contrast to the province of Los Santos. Identifying HD cases in the former regions may be stymied by the impact that the suspension of the February carnival festivities had on the social, cultural, and economic aspects of these communities. In addition, patients do not always seek medical attention for this relatively mild disease. We have previously found that the ratio of total HV infections to moderately severe HPS is 9:1, which is similar to the ratio of annual seroconversions to hospitalized HPS of 14:1 [18,42]. Based on that observation, we expected that the HD diagnoses would be higher; however, this was not observed in three of the four provinces of the endemic area. It is possible that adequate differential diagnoses were not made [25,44]. Despite the intense health promotion campaign for prevention and associated research studies carried out in the endemic area, the systematic efforts to search for patients and conduct surveys may have been inadequate [26] by the health services.

## 5. Conclusions

HD in Panama has a mild presentation, affects all age groups, and deaths have occurred in individuals over 16 years of age, although the case-fatality rate mainly affects the age group of 20 to 49 years. The lethality caused by CHOV is lower than that observed in ANDV and SNV. Although men may test positive more often than women, the HPS case-fatality rate is slightly (but not significantly) higher in women, as reported elsewhere in the Americas. Outbreaks occur every 4–5 years, and cases occur every month of the year, with peaks during the dry season and part of the rainy season, possibly influenced by agricultural activity. Decentralization of the laboratory test and dissemination of evidence-based surveillance guidelines and regulations have standardized and improved diagnosis, notification, and documentation at the level of the primary care system and management in intensive care units nationwide. We have defined an endemic area for the disease that represents 27% of the national territory; however, this does not rule out the circulation of CHOV in other areas. *Oligoryzomys costaricensis* has been captured nationwide at elevations below 700 m in areas that meet the agroecological conditions feasible for supporting those rodent populations [21]. Given the commensal association of this rodent with crops and grassland pastures, it is likely only a matter of time before this rodent expands into newly developed agricultural sites. Surveillance and investigations carried out during the last two decades have allowed us to learn about this endemic zoonotic disease, but much remains to be elucidated. We are currently conducting research to determine the immunogenetic profile, to further examine rodent population dynamics through long-term site-intensive field studies to better understand their ecology, and to design interventions within a One Health approach to generate more efficient and rapid diagnostic techniques to aid in treatment, prevention and, ultimately, mitigation HD’s effects on the Panamanian population.

## Figures and Tables

**Table 1:** Demographic characteristics and spectrum of hantavirus disease by province, 1999–2019.

Characteristics	Endemic Area					
	Coclé	Herrera	Los Santos	Veraguas	Panamá	Total
n	49	22	550	84	7	712 ^#^
Male sex (%)	57.1	45.5	55.6	64.3	57.1	56.5
Mean age (±SD) years	40.2 (±15.0)	45.3 (±15.9)	36.3 (±19.1)	38.6 (±17.1)	45.6 (±16.4)	37.1 (±18.5)
Age group						
<18 years	3	0	103	10	1	117
≥18 years	46	22	447	74	6	595
Spectrum						
Hantavirus fever	9	2	234	21	0	266
<18 years	0	0	73	6	0	79
≥18 years	9	2	161	15	0	187
Hantavirus pulmonary syndrome	40	20	316	63	7	446
<18 years	3	0	30	4	1	38
≥18 years	37	20	286	59	6	408
Deaths	12 *	4 *	29	8	3	56
<18 years	2	0	0	0	1	3
≥18 years	10	4	29	8	2	53

^#^ One patient, whose onset of illness was 24 August 1999, was identified as retrospective [13]. * *p* < 0.05 with respect to the cumulative case-fatality rate of Los Santos (5.3%, Herrera = 24.5%; Coclé = 18.2%).

## Data Availability

The data presented in this study are available on request from the corresponding author.

## Supplementary Materials

The following supporting information can be downloaded at: https://www.mdpi.com/article/10.3390/v15061395/s1, Figure S1: (a) Spectrum of hantavirus disease in Panama, 1999–2019. HD = Hantavirus disease. HF = Hantavirus fever. HPS = Hantavirus Pulmonary Syndrome. In 249 negative samples for hantavirus, no other cause was specified. IgM antibodies against DENV were detected in fifteen HD patients (HF: 4, HPS: 11). Case-fatality rate= 12.6% (56/446); One patient, whose onset of illness was 24 August 1999, was identified retrospective [13]; (b) Definitions of four HV disease categories [18]; Figure S2. Seven cases of HPS reported in the Province of Panama, 1999–2019. The green squares with the numbers correspond to the cases of HPS diagnosed per year; Figure S3. Number of mild (HF + HPS mild) and severe (HPS moderate + HPS severe) cases of hantavirus disease per year, 1999–2019.
