# Supplementary material for: Hantavirus in Panama: Twenty Years of Epidemiological Surveillance Experience
Source: Viruses. 2023 Jun 19;15(6):1395. doi: 10.3390/v15061395 (PMC10304491; doi:10.3390/v15061395)
Supplement: Supplementary file 1 [file viruses-15-01395-s001.zip › viruses-2428505-supplementary.pdf]

(**a**)

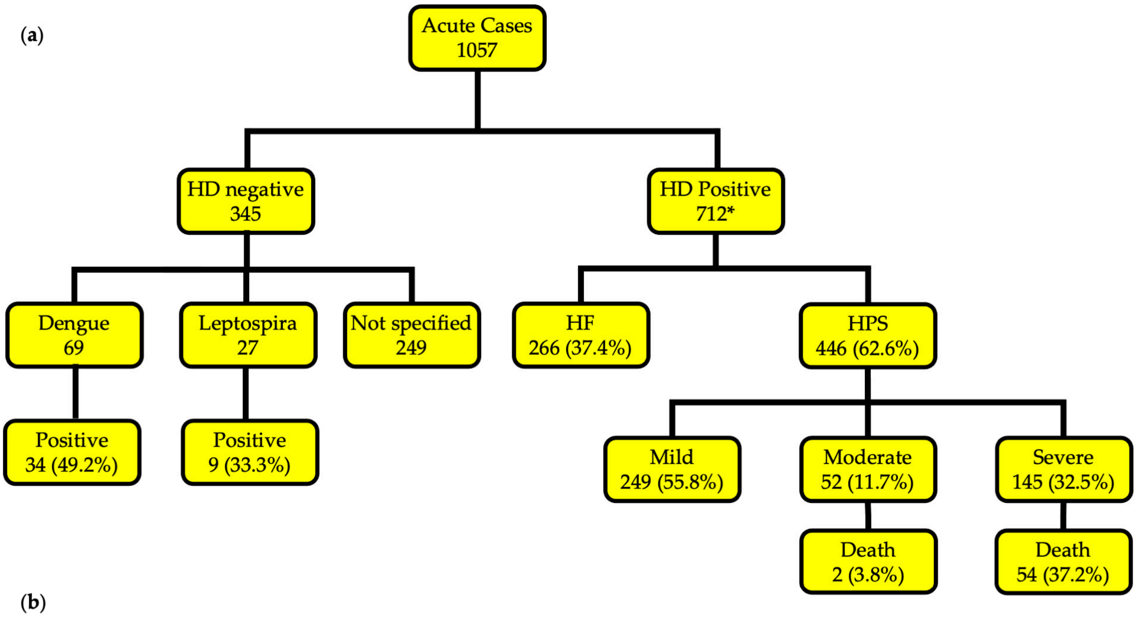

(**b**)

Criteria	Categories			
	Hantavirus fever	Mild HCPS	Moderate HCPS	Severe HCPS
**Febrile prodome**	+	+	+	+
**Dyspnea***	Absent	Absent or occasional	Marked	Marked
**Chest X-ray**	Normal	Abnormal	Abnormal	Abnormal
**Hypoxemia**	Absent	SaO_2 >85%	SaO_2 <85%	SaO_2 <85%
**Oxygen therapy**	No	Nasal cannula or face mask	CPAP/MV	MV + inotropic/vasopressor

Figure S1: (a) Spectrum of hantavirus disease in Panama, 1999–2019. HD = Hantavirus disease. HF = Hantavirus fever. HPS = Hantavirus Pulmonary Syndrome. In 249 negative samples for hantavirus, no other cause was specified. IgM antibodies against DENV were detected in fifteen HD patients (HF: 4, HPS: 11). Case-fatality rate= 12.6% (56/446); One patient, whose onset of illness was 24 August 1999, was identified retrospective [13]; (b) Definitions of four HV disease categories [18].

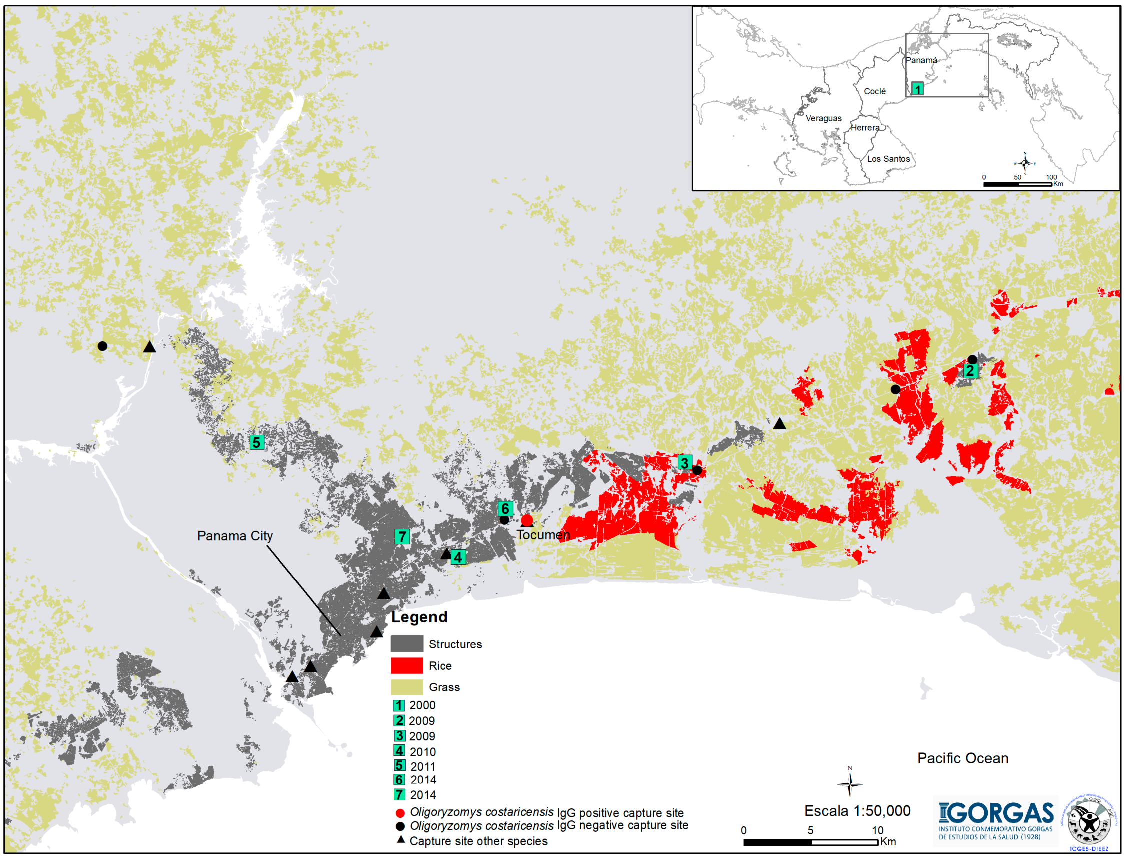

Figure S2. Seven cases of HPS reported in the Province of Panama, 1999–2019. The green squares with the numbers correspond to the cases of HPS diagnosed per year.

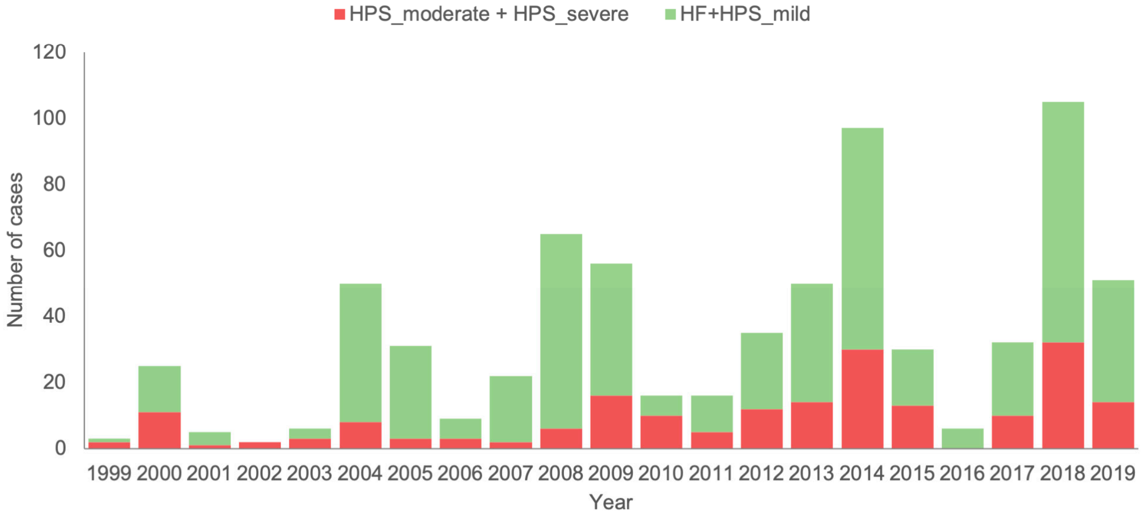

Figure S3. Number of mild (HF + HPS mild) and severe (HPS moderate + HPS severe) cases of hantavirus disease per year, 1999–2019.
