# Supplementary figures and images for: Hantavirus in Panama: Twenty Years of Epidemiological Surveillance Experience
Source: Viruses. 2023 Jun 19;15(6):1395. doi: 10.3390/v15061395 (PMC10304491; doi:10.3390/v15061395)

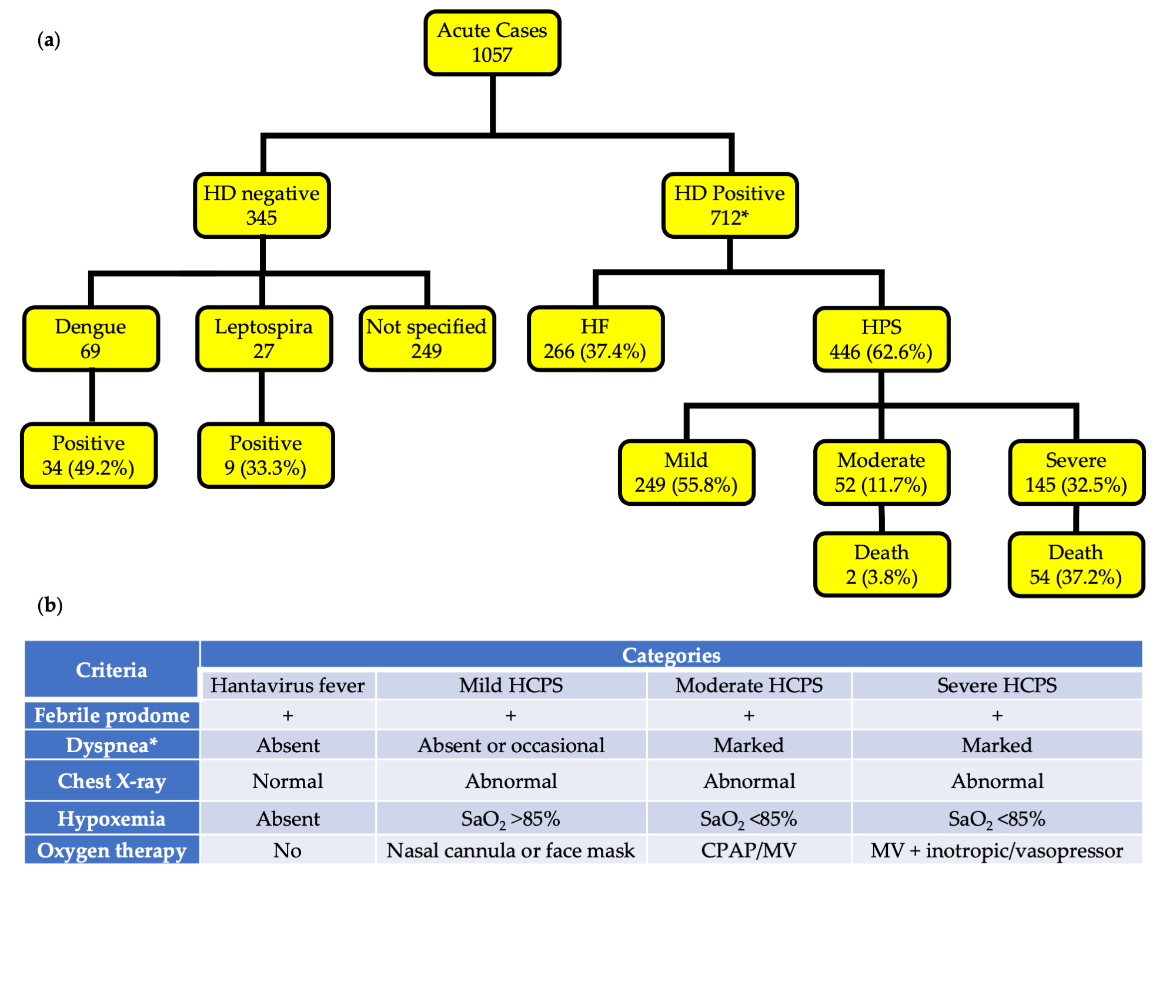

Supplement: Supplementary file 1 [file viruses-15-01395-s001.zip › viruses-2428505-supplementary/Figure S1_16052023.tiff]

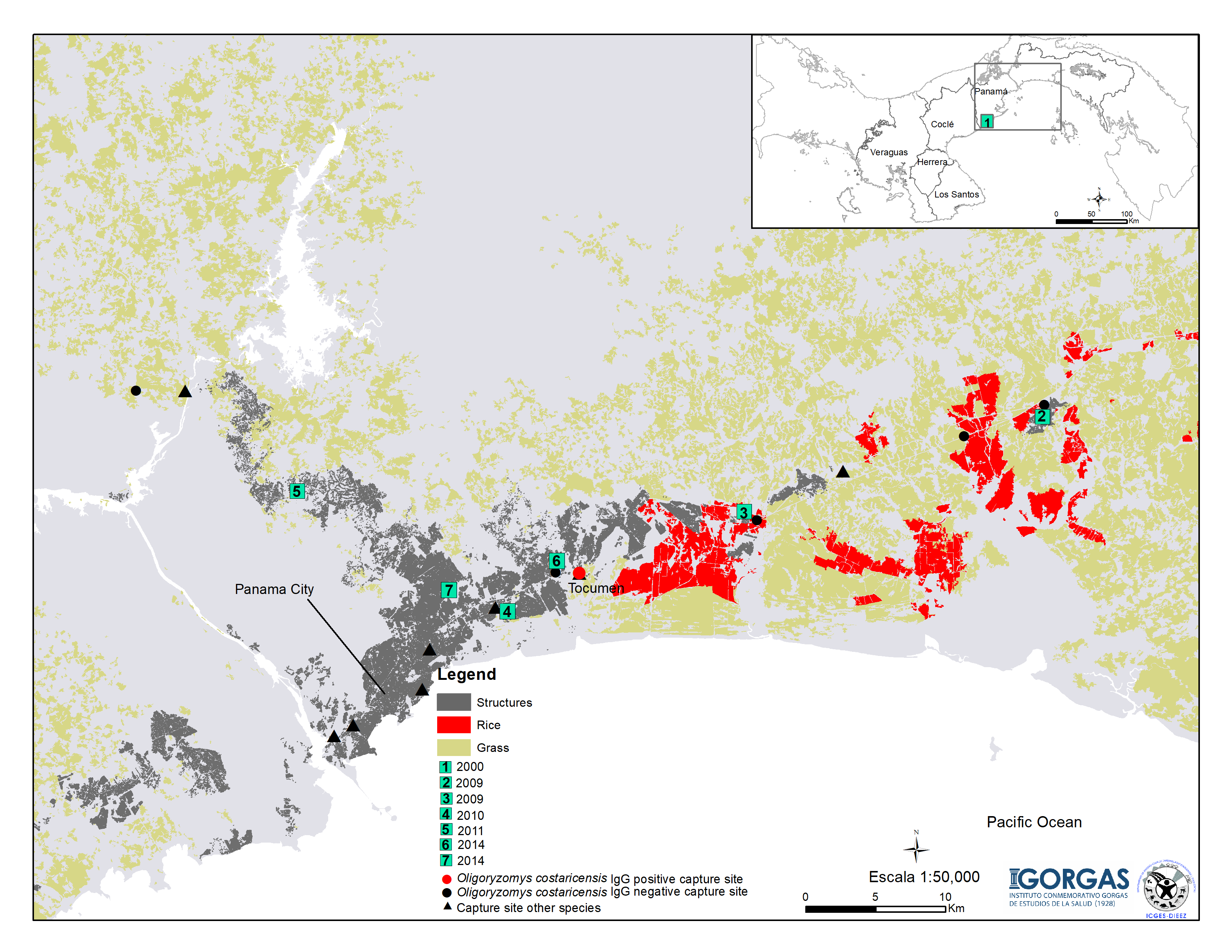

Supplement: Supplementary file 1 [file viruses-15-01395-s001.zip › viruses-2428505-supplementary/Figure S2_06062023.tif]

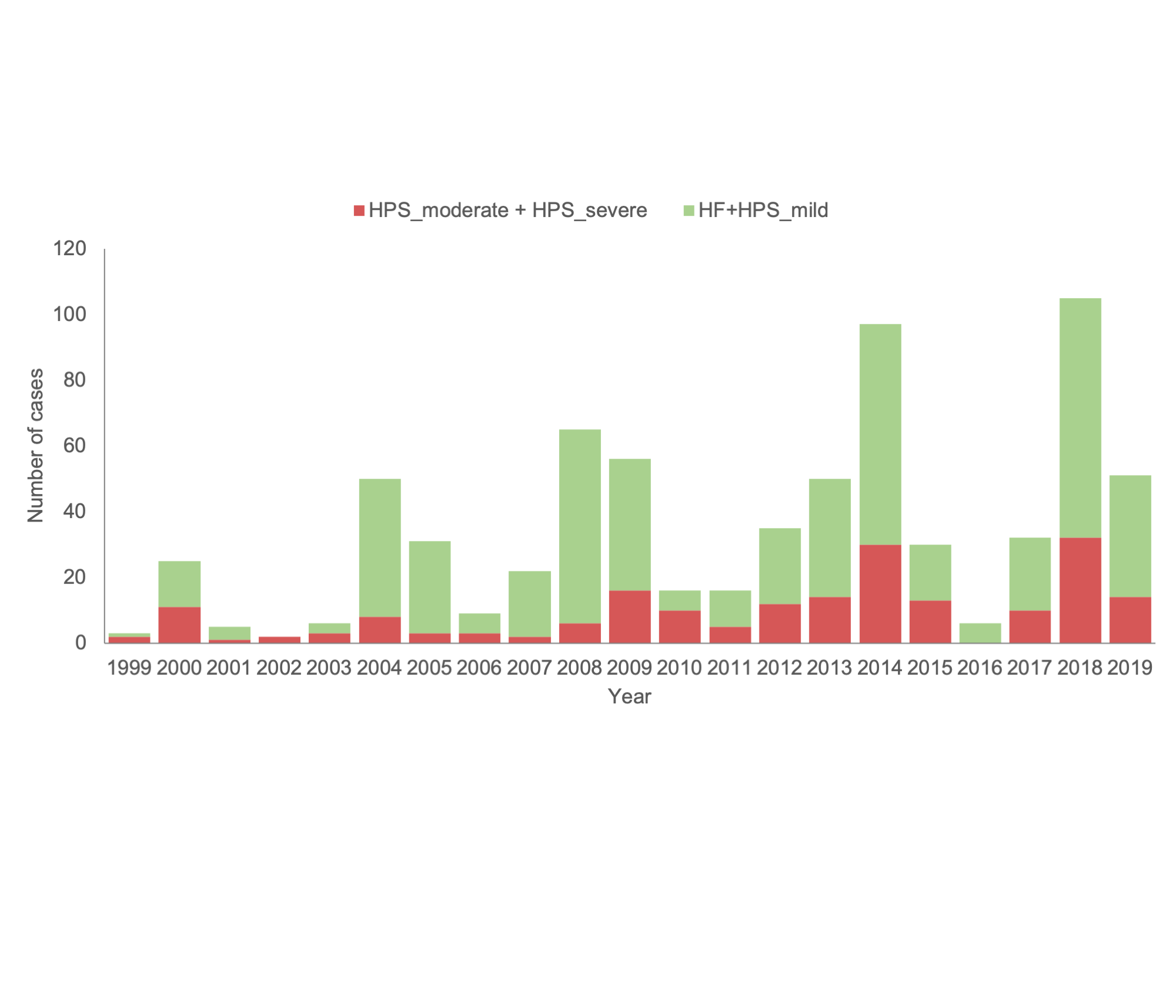

Supplement: Supplementary file 1 [file viruses-15-01395-s001.zip › viruses-2428505-supplementary/Figure S3_16052023.tiff]
